# Establishment of a Simple and Rapid Nucleic Acid Detection Method for Hookworm Identification

**DOI:** 10.3390/pathogens12040630

**Published:** 2023-04-21

**Authors:** Xin Ding, Yougui Yang, Yingshu Zhang, Qiang Zhang, Fanzhen Mao, Yang Dai

**Affiliations:** 1 Key Laboratory of National Health Commission on Parasitic Disease Control and Prevention, Key Laboratory of Jiangsu Province on Parasite and Vector Control Technology, Jiangsu Institute of Parasitic Diseases, Wuxi 214064, China; 2 School of Public Health, Nanjing Medical University, Nanjing 211166, China

**Keywords:** hookworm identification, recombinase-aided amplification, *Ancylostoma duodenal*, *Necator americanus*, Kato-Katz, larvae culture

## Abstract

Hookworm infection is one of the most common neglected tropical diseases and is mainly found in tropical and subtropical areas. Two species of human hookworm are distributed in China, i.e., *Ancylostoma duodenale* (AD) and *Necator americanus* (NA). Background: Traditional microscopic technology such as the Kato-Katz method is not suitable for hookworm diagnosis due to the rapid degeneration of fragile hookworm eggs or for species identification of hookworm infection. The aim of the present study was to establish and evaluate a novel nucleic acid detection method based on recombinase-aided isothermal amplification (RAA) for the detection of hookworm infections and species identification. Methods: Based on the specific target gene sequences of hookworms (*5.8S rRNA* for AD and *ITS2* for NA, respectively), we designed and synthesized amplification primers and fluorescence probes referring to the principle of the fluorescence recombinase-aided amplification (RAA) technique. Results: Each assay provided specific amplification of larval DNA from AD and NA by fluorescence RAA, and the detection limits in plasmids reached 10^2^ copies and 10 copies, respectively. Genomic DNA of two hookworm species was successfully detected at a concentration of 0.1 pg/μL, revealing a high detection sensitivity. No positive amplification occurred for genomic DNA from crossed hookworm species and genomic DNA from *Cryptosporidium*, *Giardia lamblia*, *Strongyloides stercoralis*, *Schistosoma japonicum*, *Ascaris lumbricoides*, and *Clonorchis sinensis*, revealing a satisfactory specificity. Fecal sample detection results demonstrated a similar efficacy to the Kato-Katz method; however, it had a greater sensitivity than the larvae culture method. Conclusion: A simple and rapid nucleic acid method was successfully established based on RAA, which improved the detection efficacy and species identification for human hookworm infections.

## 1. Introduction

Hookworm infection is one of the most common neglected tropical diseases and is mainly found in tropical and subtropical areas. It affects nearly 471.8 million people worldwide, causing dysfunction in the digestive tract and iron deficiency anemia [1,2]. The infection rate of hookworm disease in China is 2.62%, and the estimated number of infections is about 16.9 million, according to the Report on the National Survey of Important Human Parasitic Diseases in 2015, causing a substantial economic and health burden [3,4]. Although the rates of hookworm infection have been further reduced and it is considered a predominantly mild infection at present in China due to years of continuous prevention and control efforts [4,5], hookworm infection monitoring is still needed. Two species of hookworm, i.e., *Ancylostoma duodenale* (AD) and *Necator americanus* (NA), are considered to be the predominant species infecting humans in China. These two species have a different geographical distribution [6,7]; also, the degree of anemia severity differs between the two species, as AD infection can cause more severe blood loss and iron deficiency anemia compared with NA [8]. Species identification for human hookworm infection is essential for prevention and treatment in the regular monitoring work.

An accurate diagnosis of hookworm infection is crucial in formulating effective prevention and control strategies such as large-scale deworming. The present diagnosis techniques still predominately rely on etiological techniques, including the Kato-Katz and larvae culture methods. The Kato-Katz method is one of the most commonly used methods for diagnosing and monitoring soil-transmitted helminth (STH) infections due to its operational portability and relatively low cost in most rural areas [9]. However, its detection sensitivity decreases for infections of really mild intensity [10]. Additionally, species identification cannot be achieved by this method for the same egg morphology of two species. Furthermore, it loses efficacy for hookworm infections due to the rapid degeneration of fragile hookworm eggs over two hours [11,12]. Moreover, the laborious process results in the poor performance of the Kato-Katz method for hookworm detection [13]. The larvae culture technique is a conventional measure used for species identification of hookworm through different filariform larvae morphology of two species. However, its successful outcome largely relies on the survival of larvae isolated from stool samples and skillful operators [14,15]. Accordingly, novel detection methods with high sensitivity and specificity for hookworm infection detection and species identification are urgently needed.

In recent years, techniques based on nucleic acid amplification for parasite infection detection, such as the polymerase chain reaction (PCR), have been developed [16], revealing a higher sensitivity and specificity compared with the Kato-Katz method for hookworm detection in the case of low-intensity infections [17,18]. Yet, the requirements for sophisticated equipment and laboratories and professional, well-trained operators could hamper the field detection ability in hookworm infection monitoring [19]. Recombinase-aided amplification (RAA), as an isothermal amplification technique, uses a recombinant enzyme, a single-strand binding protein, and DNA polymerase to achieve efficient amplification of nucleic acid within 5–20 min under isothermal conditions (25–42 °C), offering the advantages of convenient operation and a short reaction time. It does not require a thermal cycle and longer time, as do conventional nucleic acid amplification techniques [20], and in recent years, it has been successfully applied in the detection of a variety of pathogens, including viruses and bacteria [21,22]. Meanwhile, various RAA detection methods have been established and evaluated for parasites, including *Schistosoma japonicum*, *Clonorchis sinensis*, *Echinococcus granulosus*, *Angiostrongylus cantonensis*, and related parasites, revealing a considerable sensitivity and specificity [23,24,25]. The rapid detection, simple operation, and high efficiency make this method a more advantageous choice as a detection tool.

Therefore, the aim of the present study was to establish a novel nucleic acid detection method based on the recombinase-aided isothermal amplification (RAA) technique and evaluate its capacity for hookworm infection detection and species identification by using laboratory and human fecal samples, thus providing a feasible detection tool for future hookworm infection monitoring.

## 2. Materials and Methods

### 2.1. Parasites and DNA Samples

Parasite samples used in the present study included metacercariae of *Clonorchis sinensis*, larvae and eggs of AD and NA, cysts of *Giardia lamblia*, oocysts of *Cryptosporidium*, larvae of *Strongyloides stercoralis*, and eggs of *Schistosoma japonicum* and *Ascaris lumbricoides*, which were collected and stored in Jiangsu Institute of Parasitic Diseases. Different kits were used for genomic DNA extraction, corresponding to different types of parasite samples. Genomic DNA from cysts of *Giardia lamblia*, oocysts of *Cryptosporidium*, metacercariae of *Clonorchis sinensis*, and larvae of AD and NA were extracted by DNeasy Blood and Tissue Kits (Qiagen, Hilden, Germany). Genomic DNA from the eggs of *Schistosoma japonicum*, *Ascaris lumbricoides*, AD, and NA were extracted by QIAamp PowerFecal DNA Kits (Qiagen, Germany). All the operations were carried out according to the protocol of the kit, following a determination by Thermo NanoDrop 2000 (ThermoFisher, Waltham, MA, USA); all extracted genomic DNA samples were stored at −80 °C.

### 2.2. Target Sequence Screening and Primer Design

The whole gene sequences of *5.8S rRNA* of AD and *ITS2* of NA were searched and obtained from the National Center for Biotechnology Information (NCBI) Database Genebank (accession number: EU344797.1, 1 December 2007 and Y11734.1, 6 March 1997) [26,27]. Multiple sequence alignment and homology analyses were carried out by DNAMAN 7.0 software. A conservative sequence of each species was selected as the target sequence for the RAA reaction. Primers and probes were designed by Amplfix software and were further validated to avoid nonspecific similarity of sequences. All of the primers, probes, and plasmids used in this study were synthesized by Sango Biotech (Shanghai, China). The details of the primers and probes are listed in Table 1.

### 2.3. Establishment of an RAA Reaction System

The basic RAA reaction system (50 μL) included 25 μL of rehydration buffer, 2 μL of forward primer (0.1 mmol/L), 2 μL of reverse primer (0.1 mmol/L), and 17.5 μL of double-distilled H_2_O (ddH_2_O) that were briefly vortexed, after which 2.5 μL of magnesium acetate solution (280 mmol/L) was added and 1 μL of hookworm genomic DNA (30 ng/L) was used as a template. Subsequently, the above reaction system was added to the enzyme mixture (SSB, 800 ng/μL; UvsX and UvsY protein, 120 ng/μL; and DNA polymerase, 30 ng/μL) and rapid centrifugation was performed after dissolution. The tube lid was kept closed and transferred into a sample pretreatment system RAA-B6100 (Jiangsu Qitian Bio-tech Co., Ltd., Wuxi, China) and incubated at 37 °C for 30 min. After the reaction, 50 μL 1:1 phenol/chloroform was added to the amplification products for DNA extraction, then centrifuged at 12,000× *g* for 1 min. Then, 10 μL of upper layer solution was drawn and analyzed by agarose gel electrophoresis (1%, 100 V, 60 min). In addition, the amplification products were checked under an ultraviolet lamp and sequenced by Sango Biotech (Shanghai, China).

The fluorescence RAA reaction system (50 μL) included 25 μL rehydration buffer, 3 μL upstream and downstream primers mixer (0.05 mM), 1 μL fluorescence probe (0.02 mM), 17.5 μL dd H2O, 2.5 μL magnesium acetate solution (280 mmol/L), 1 μL DNA template to be tested, and the lyophilized RAA (including exonuclease) enzymes in a thin 0.2 mL tube. Each test required the setting of negative (dd H_2_O) and positive (recombinant DNA plasmid of the target gene of AD and NA) references. The above reaction system was vortexed in a sample pretreatment system (RAA-B6100) and then transferred to an isothermal nucleic acid amplification detector (RAA-F1620) (Jiangsu Qitian Bio-tech Co., Ltd., Wuxi, China) at 37 °C for 20 min. The fluorescence value was recorded every 20 s. According to the positive judgment method of the amplification detector, the *K* value (the slope value of fluorescence signal in the rising stage) was used to further determine whether the increase in fluorescence signal was a true change. If the slope of the fluorescence signal detected at consecutive time points exceeded the defined level, it was considered a “positive” signal and vice versa. In this study, we defined the *K* value as 20 so that *K* ≥ 20 was positive and *K* < 20 was negative.

### 2.4. Sensitivity and Specificity Evaluation

For sensitivity evaluation, minimum copy numbers of target sequences and the concentration of genomic DNA (AD or NA) were used for fluorescence RAA reactions. The target sequence from AD and NA was subcloned into a plasmid vector (pUC57) and subsequently transformed into *Escherichia coli* (DH5 α). After culture and extraction, the concentration of recombinant plasmids was determined and different copy numbers of the plasmid were obtained through gradient dilution, which included 10, 10^2^, 10^3^, 10^4^, 10^5^, 10^6^, and 10^7^ copies/μL. Genomic DNA from hookworm (extracted previously) was diluted to 10 ng/μL and conducted to obtain concentrations of 10, 1, 0.1, 0.01, and 0.001 ng/μL DNA samples through gradient dilution. For specificity evaluation, genomic DNAs from *Cryptosporidium*, *Giardia lamblia*, *Strongyloides stercoralis*, *Schistosoma japonicum*, *Ascaris lumbricoides*, *Clonorchis sinensis*, AD, and NA were used as templates to evaluate the specificity of present RAA methods. A total of 5 μL of each DNA sample (recombinant plasmids with different copy numbers, hookworm genomic DNAs with different concentration, and genomic DNAs from other different parasites) was taken as a template for RAA amplification. The positive judgment was the same as the fluorescence RAA reaction system mentioned above. Each sensitivity assessment was repeated at least six times and the specificity assessment was repeated at least three times.

### 2.5. Comparison of Detection Efficacy for Human Fecal Samples Using Different Methods

A total of 206 human fecal samples were collected from rural areas in the northern part of Jiangsu Province, China, and used to compare the detection capacity with different methods. Each stool sample was examined with the triplicate Kato-Katz method to detect hookworm eggs according to the standard procedure [28]. The counts of eggs per gram (EPG) were obtained by calculating the egg quantity from each triplicate [29]. DNA from all human fecal samples was extracted by a QIAamp PowerFecal DNA Kit (Qiagen, Germany) according to the kit instructions and used as the template for fluorescence RAA detection with the same reaction conditions and positive judgment mentioned above. Larvae culture of positive stool samples detected by the Kato-Katz method was carried out according to the Harada-Mori culture method [14], and each sample was daubed on a double test strip to avoid operational errors. The horizontal grain tunica vaginalis and oral spear of cultured larvae were examined with microscopy and used for morphological hookworm species identification. The RAA detection efficacy for hookworm infection was evaluated through human stool samples with various infection intensities and compared with the other two methods. The Kato-Katz and larvae culture methods were also compared for their detection efficacy, and the fluorescence RAA and larvae culture method were compared for their species identification capacity.

### 2.6. Ethics Statement

The study protocol was approved by the Ethical Review Committee of the Jiangsu Institute of Parasitic Diseases (JIPD-2021-010). All participants were informed of the purpose of this study and gave their informed consent. A total of 206 stool samples were provided voluntarily by participants and were transported under refrigerated conditions to the laboratory. All patients who were positive for hookworm eggs or larvae were treated with oral albendazole (GlaxoSmithKline, Brentford, UK); regular follow-ups and retesting were conducted.

### 2.7. Statistical Analysis

The statistical analyses were performed using IBM SPSS Statistics, version 22 (IBM Corporation, Armonk, NY, USA). The detection results between three methods were compared by the kappa value (κ).

## 3. Results

### 3.1. The RAA-Based Method Was Successfully Established for Hookworm Detection

**Figure 1:**
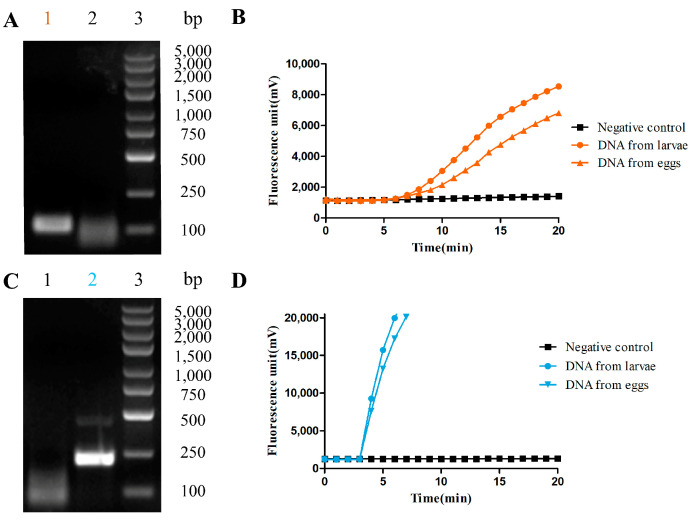
Results of RAA methods for hookworm DNA samples. (**A**) RAA agarose electrophoresis of AD genomic DNA. 1 is AD, 2 is negative control, 3 are MDL-5000 DNA markers; (**C**) RAA agarose electrophoresis of NA genomic DNA. 1 is negative control, 2 is NA, 3 are MDL-5000 DNA markers; (**B**,**D**) the fluorescent RAA amplification of hookworm larvae DNA extracts from AD and NA, respectively (*n* ≥ 3).

To test the efficacy of the selected target sequence, basic RAA reaction systems were established. Genomic DNA from two species of hookworm was first conducted as a template for a basic RAA reaction. As shown in Figure 1A,C, the molecular weights of amplified fragments of AD and NA genomic DNA observed under the UV lamp by 1% agarose gel electrophoresis were around 116 bps and 219 bps, respectively. The sequencing results by Sango Biotech showed homology with the target sequences.

To perform real-time detection, fluorescence RAA reaction systems were further established. Genomic DNA from two hookworm species was used as a template and a fluorescence probe was designed (Table 1). The mixed reaction tube was placed into the fluorescence detector and was incubated at 37 °C for 20 min with a signal obtained every minute. The results showed that two DNA samples from AD had positive amplification at 6 min with *K* values of 876 and 523, respectively (Figure 1B). Additionally, DNA samples from NA had positive amplification at 3 min with *K* values of 5562 and 6304, respectively (Figure 1D). No amplification was observed in any of the two negative controls, which indicated that both target sequences could be detected and that the fluorescence RAA method was successfully established for AD and NA.

### 3.2. Thr Fluorescence RAA-Based Method Showed High Sensitivity and Specificity for Hookworm Detection

**Figure 2:**
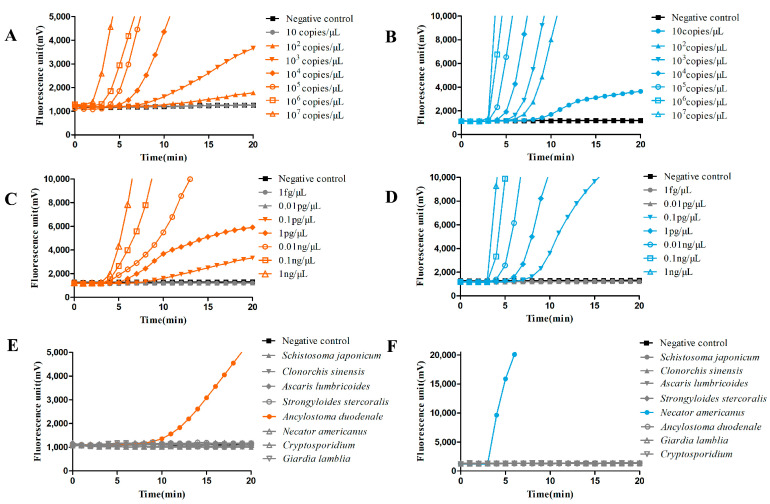
Results of sensitivity and specificity evaluation for RAA. (**A**,**B**) Sensitivity evaluations for the RAA detection of recombinant plasmids with different copy numbers from AD and NA, respectively (*n* ≥ 6). (**C**,**D**) Sensitivity evaluations for RAA detection of different concentrations of DNA from AD and NA, respectively (*n* ≥ 6). (**E**,**F**) Specificity evaluations for RAA detection of AD and NA compared with other kinds of parasites, respectively (*n* ≥ 3).

To evaluate the sensitivity, recombinant plasmids containing different gradient copy numbers of genomic DNA from AD and NA were detected by fluorescence RAA. Each sample could achieve positive amplification up until about 10 min, and their *K* value was delayed along with the decline in the copy number. The *K* values observed from 10^7^ copies/μL to 10^2^ copies/μL of AD plasmid were 2826, 1219, 1028, 367, 229, and 124, respectively. No signal was found at 10 copies/μL from AD (Figure 2A). The *K* values of NA plasmid were 6974, 5871, 1362, 569, 478, 325, and 116 from 10^7^ copies/μL to 10 copies/μL, respectively (Figure 2B). It was suggested that the sensitivity of fluorescence RAA could reach 10^2^ copies/μL for AD and 10 copies/μL for NA using recombinant plasmid as a template. Meanwhile, we further evaluated genomic DNA from two species with different concentrations by fluorescence RAA, finding that the DNA samples from AD with concentrations from 1 ng to 0.1 pg/μL could be successfully amplified, and those with 0.01 pg/μL and 1 fg/μL failed to detect any signal (Figure 2C). Similar results were observed for NA genomic DNA amplification (Figure 2D), which indicated that the detection sensitivity could reach 0.1 pg/μL for two hookworm species using genomic DNA as a template.

To evaluate the specificity, the genomic DNA from *Cryptosporidium*, *Giardia lamblia*, *Strongyloides stercoralis*, *Schistosoma japonicum*, *Ascaris lumbricoides*, and *Clonorchis sinensis* was used as a template for fluorescent RAA. For the AD RAA reaction system, only the AD genomic DNA sample showed positive amplification at 10 min with a *K* value of 543, while no signal was observed from other parasite samples (Figure 2E). Additionally, only the NA genomic DNA sample showed positive amplification at 5 min, with a *K* value of 5817 for the NA RAA reaction (Figure 2F). Furthermore, there were no cross-reactions between the two hookworm species for the two RAA reaction systems. The results indicated a considerable specificity of the RAA method for AD and NA, respectively.

### 3.3. The Fluorescence RAA-Based Method Showed a High Detection Capacity of Hookworm Infection for Human Fecal Samples

In order to assess the detection capacity for human samples, 206 fecal samples were collected from the northern area of Jiangsu Province, China, and were detected by the fluorescence RAA, Kato-Katz, and larvae culture methods, respectively. As shown in Table 2, 77 (positive rate 37.38%) and 73 (positive rate 35.44%) positive samples were detected among 206 samples by the RAA and Kato-Katz methods, respectively. Four conflicting samples all occurred in the low-density range of EPG (below 500, shown in Table 3) and were proven positive when confirmed by further complementary detection using the Kato-Katz method. For the larvae culture method, only 48 fecal samples (positive rate of 23.30%) were positive, and only high EPG samples (>4000) coincided with the other two methods. It was found that missing inspection occurred for the culture method, especially for mild infection in human fecal samples. These results suggest that the fluorescence-RAA-based method has an approximate detection efficacy compared with the Kato-Katz method and is more sensitive than larvae culture for hookworm infection detection.

### 3.4. The Fluorescence RAA-Based Method Could Be Applied to Hookworm Species Identification in Human Fecal Samples

**Figure 3:**
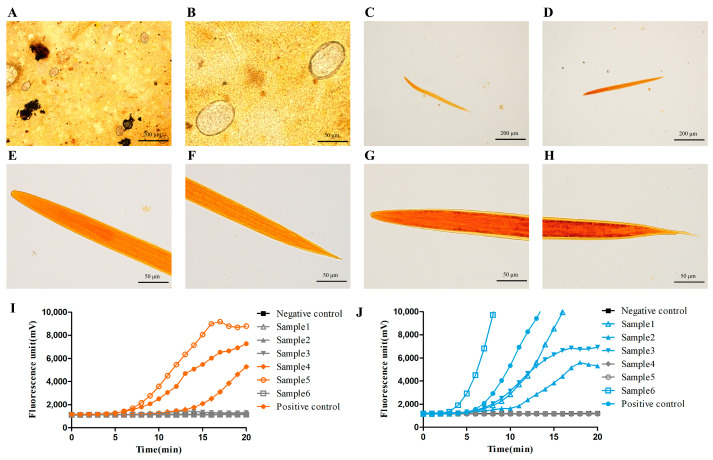
Morphological characteristics of hookworm eggs and larvae as well as the results of RAA methods for field samples. (**A**,**B**) Morphological characteristics of hookworm eggs observed under an optical microscope a few hours after Kato-Katz operator. (**C**,**E**,**F**) Morphological structure under iodine staining of larvae produced from stool culture and identified as AD. (**D**,**G**,**H**) Morphological characteristics of NA. (**I**,**J**) One group from the field sample validation by RAA for AD and NA, respectively (*n* ≥ 3).

As shown in Figure 3A,B, there were no morphological differences in hookworm eggs determined by microscopy, and species identification needed to be further carried out through other methods, such as larvae culture. Following positive verification through RAA and Kato-Katz methods, 77 stool samples were applied and used for species identification. For the larvae culture method, culture larvae were observed in only 48 egg-positive stool samples, while detection failed in a substantial number of fecal samples, especially those with mild and medium infection intensities (below 4000 of EPG), which indicated the lower detection efficiency of larvae culture for mild and medium intensity EPG levels compared with RAA (Table 3). As shown in Table 4, these 48 positive samples were further classified into 2 samples of AD infection, 44 samples of NA infection, and 2 samples with mixed infection, which were identified through larvae morphological characteristics, including horizontal grain tunica vaginalis and oral spear, as shown in Figure 3C–H. However, all 77 samples were successfully detected and synchronously classified into different species through the fluorescence RAA method, including 5 samples of AD infection, 65 samples of NA infection, and 7 samples with mixed infection (Figure 3I,J). These results indicated that fluorescence RAA could be used for species identification in hookworm infection.

## 4. Discussion

In the present study, we established a novel RAA-based detection method, which showed a satisfactory efficacy for hookworm infection detection and species identification, as was evaluated with laboratory and human fecal samples. It should be noted that the primer and probe corresponding to the target sequence we designed for AD in this research has a high homology with *Ancylostoma ceylanicum* (*A. ceylanicum*). Based on the convenience of the construction of the RAA system and the low incidence rate of infection of *A. ceylanicum* in Jiangsu areas [4], we finally selected *5.8S rRNA* as a target sequence of AD. Theoretically, the cross-reactivity to *A. ceylanicum* of the AD RAA system might be a weakness in our study, but there should be no doubt about identifying human infection of AD or NA in the Jiangsu area. The verification with other closely related species of *Ancylostoma* still needs to be completed. Due to no need for thermocycling in the present method, nearly all the positive amplifications could be observed in real time, i.e., within 20 min. Furthermore, compared with traditional PCR, the instrument used for operation is more convenient for transfer, and there are no other complicated procedures, thus making it easier to be used in poor conditions such as those commonly found in remote regions [20,21].

The internal transcribed spacer regions (ITS) contain highly conserved segments on both sides and internal characteristic sequences that make it widely used in PCR primer design [30]. The lower mutation rates also make it a useful marker for the identification of various species [26]. The convenience of a real-time PCR method coupled with a high-resolution melting curve analysis designed based on *ITS2* has been well confirmed in the application of diagnosis and identification for several human hookworms, with its lowest dilution reaching 0.01 ng/μL [31]. In this assay, we demonstrated the relatively high sensitivity of the RAA reaction system compared to PCR, where the detection limits of the genomic concentration were at least 0.1 pg/μL for both species. According to the practical detection performance for human stool samples, RAA revealed more satisfactory sensitivity than Kato-Katz, especially when the infection was very low. It should be noted that Kato-Katz is not applicable to differentiate the species due to the morphological similarity between AD and NA under an ordinary optical microscope [32]. Thanks to the continuous decline in hookworm infection rates in Jiangsu over recent years [5], the comparison between Kato-Katz and RAA methods still needs to be further explored by a follow-up abundant specimen verification.

Previous studies have reported that different hookworm species could develop relevant drug resistance mechanisms, which is why species identification is crucial for prevention and treatment strategies [27,33,34]. However, misdiagnosis often occurs in larvae culture when the eggs fail to develop, even not to say the effort it consumes and high technical requirements for microscopists [16,35]. We observed that some larvae obtained from stool cultures failed to hatch into the characteristic third stage in our assay, which was disadvantageous for subsequent identification. In fact, during the observation process, larvae may be at different stages of development, coupled with differences in their movement, light, and angle, which may lead to confusion and neglect. Currently, molecular identification techniques, including conventional PCR and real-time PCR, which are more specific and reliable than traditional morphologic methods, are still the mainstream methods for species differentiation [36]. These methods involve conducting a specific amplification through various target sequences so as to achieve diagnosis and evaluation based on different hookworm species. They are easier to use for large-scale screening of long-term population monitoring [37]. In the present study, the detection effect of larvae culture was unstable for infections of a moderate degree and poor for infections of low intensity. However, the RAA method showed a considerable positive detection rate compared with larvae culture and could be applied as a pattern to distinguish species. Furthermore, the proposed RAA assay could effectively identify the infection types of the hookworm disease, including simple and mixed infections.

To sum up, based on the RAA technique platform, we successfully achieved rapid and efficient amplification for hookworm infection, which showed high sensitivity and specificity compared with the Kato-Katz and larvae culture methods, providing a viable choice for the future hookworm infection monitoring. However, the present study has some limitations. Firstly, two separated units of the RAA reaction system were established for AD and NA detection. Thus, running both units for one specimen is necessary to distinguish the infection and species identification. Additionally, integration of the two units into one RAA reaction system might improve the detection efficacy for hookworm infection and species identification. Secondly, a comparison of the detection efficacy with RAA and other molecular detection technologies needs to be further explored in future studies. Finally, the limitation of cross-reactivity to closely related species of *Ancylostoma* also needs further attention and verification.

## Figures and Tables

**Table 1:** Sequences of primers and probes for RAA.

Sequences	AD (5′-3′)	NA (5′-3′)
Forward primer	CGATACGCGAATCGACCGATCCATCGCTGAAG	CTGTTTGTCGAACGGTACTTGCTCTGTACTACG
Reverse primer	ATCTGCTAACGCGGACGCCAGTACAGCAATAAC	TCCGTTCAACCACGCTCATAAGTCGCGAGAGC
Fluorescence probe ∗	TCGACCGATCCATCGCTGAAGCTAGTCGA(FAM)TT(THF)(BHQ)TGACATAAAGTCACG	TGCAACATGTGCACGCTGTTATTCACTACGT(FAM)TA(THF)(BHQ)TTRGCTAGTTTACTAAC

∗ FAM is the fluorescent reporter group, THF is tetrahydrofuran, and BHQ is the fluorescence quenching group).

**Table 2:** Results of human fecal samples detected by using the RAA, Kato-Katz, and larvae culture methods.

Results	Method		
	RAA	Kato-Katz	Larvae Culture
Negative	129	133	158
Positive	77	73	48
Kappa value	-	0.168	9.658
(vs. RAA)		(>0.05)	(<0.01)

**Table 3:** Results of human fecal samples with different EPG values detected by using the RAA, Kato-Katz, and larvae culture methods.

EPG	Method		
	RAA	Kato-Katz	Larvae Culture
1–100	21	18	7
100–500	18	17	11
500–1000	15	15	11
1000–2000	11	11	8
2000–4000	7	7	6
>4000	5	5	5
Total	77	73	48

**Table 4:** Results of species identification for hookworm-egg-positive samples by using the RAA and larvae culture methods.

Hookworm Species	Method	
	RAA	Larvae Culture
AD	5	2
NA	65	44
Mix	7	2
Total	77	48

## Data Availability

Not applicable.
